# Optimizing data linkage for maximizing the potential of Luxembourg’s national cancer registry: a comprehensive scoping review

**DOI:** 10.3389/fonc.2025.1679408

**Published:** 2025-11-18

**Authors:** Bruno Lima, Farah Hasan, Pragathy Kannan, Michael Schnell, Allini Mafra, Sophie Couffignal, Claudine Backes

**Affiliations:** 1 Registre National du Cancer du Luxembourg (RNC), Strassen, Luxembourg; 2 Cancer Epidemiology and Prevention Group (EPICAN), Department of Precision Health (DoPH), Luxembourg Institute of Health (LIH), Strassen, Luxembourg; 3 Data Integration Center, Department of Medical Information (DMI), Luxembourg Institute of Health (LIH), Strassen, Luxembourg; 4 Public Health Expertise Unit (PHE), Luxembourg Institute of Health (LIH), Strassen, Luxembourg

**Keywords:** cancer, registries, population-based register, biological specimen banks, data linkage

## Abstract

Population-based cancer registries (PBCRs) provide international standardized indicators and evaluate public health actions and cancer care. Their research potential can be significantly enhanced through linkage with secondary data sources, such as biobanks, sociodemographic or genomic data. However, legal, ethical, and technical challenges often hinder such integration. This scoping review aims at identifying data linkage opportunities between cancer registries and secondary data sources, while describing the current state of the Luxembourg’s National Cancer Registry (RNC). Ultimately, steps for linkages between cancer registries and biobanks and/or sociodemographic data are assessed to enhance cancer research and public health initiatives. A scoping review using PubMed and Embase databases was performed. English guidelines, reports, and qualitative and quantitative studies on hospital-based cancer registries, PBCRs, and site-specific registries were included. One thousand three hundred and twelve articles (n = 1312) were identified. After scanning titles and abstracts, 49 articles were examined for full-text reading, where fifteen articles met the inclusion criteria. Moreover, 13 articles were included following the snowball search approach (n = 28). Included articles report significant differences between countries in all avenues, including data availability and harmonization, confidentiality, access to data, exchange, and linkage methods. Results underline that PBCR’s potential, efficiency, and cost-effectiveness are maximized thanks to linkage activities with secondary data sources such as biobanks or sociodemographic databases. In addition, the results of this scoping review enable the identification of key questions to address before establishing data linkage grouped into five domains being: (i) legal permission, (ii) data availability assessment, (iii) data flow protocol, (iv) linkage key and (v) linkage method. In conclusion, addressing the five key domains identified in this review will support the development of robust, efficient, and ethically sound data linkage strategies, unlocking the full research potential of PBCRs and to aid decision making.

## Introduction

Cancer is a significant public health concern worldwide and has become a major barrier to increasing life expectancy (1, 2). In Europe, cancer ranks as the second leading cause of death across all age groups, resulting in substantial healthcare and social expenses (3, 4).

Luxembourg is one of the smallest European countries (672,050 inhabitants in 2024-01-01) and has the highest population growth rate (1.7%) in Europe, with foreigners representing almost half of the population (5). Cross-border workers commuting from neighboring countries (France, Belgium, and Germany) contribute to nearly half of the entire labor force. According to a 2023 Organization for Economic Co-operation and Development (OECD) report, more than one third of those covered by the Luxembourg’s National Health Insurance Fund (35.8%) are cross-border employees (6). Moreover, in countries such as Luxembourg, where the age structure of the population has changed over the last decades, with notable increase in the proportions of individuals aged 40–64 and those aged 80 and above (5), the overall cancer burden has been steadily rising over time (7). Turning the tide against cancer and reducing its economic impact is a crucial objective in today’s public health debate, as demonstrated by Europe’s Beating Cancer Plan (8). The fight against cancer needs collaborative efforts involving numerous stakeholders, including clinicians, health workforces, researchers, epidemiologists, public health professionals, and policymakers, and requires accurate and comprehensive cancer data and registration (9).

Cancer registration is a continuous process of systematic, exhaustive, and non-redundant data collection, storage, analysis, interpretation, and reporting of cancer occurrences and characteristics (10). The World Health Organization (WHO) states that population-based cancer registries (PBCRs) are a core component of cancer control strategies and the gold standard for cancer surveillance in defined communities (11). Broadly speaking, population data must be collected in a uniform and systematic way, maintaining personalized information that will allow data from different sources to be consolidated into a record per patient to be followed over time. Furthermore, the usefulness of this data depends largely on its quality, i.e. its validity, comparability, timeliness and completeness (12, 13).

PBCRs are essential for describing cancer burden, examining cancer trends, and evaluating prevention measures and cancer care. For decades, PBCRs have been supporting countries in their actions against cancer by reflecting the most representative real-world cancer situation in a well-defined geolocation. They provide internationally standardized indicators, including incidence, prevalence, mortality, and survival rates and evaluate public health interventions such as prevention, screening, and quality of care (14). Systematic assessment of these cancer indicators provides reliable information for evidence-based science policies, support policies to minimize inequalities and improve healthcare (15). The International Agency for Research on Cancer (IARC) highlights the significance of PBCRs as crucial resources for developing and evaluating cancer control plans, as well as for enhancing epidemiological and clinical research and informing cancer-related health policies.

The Luxembourg’s National Cancer Registry (in French*: Registre National du Cancer du Luxembourg*, RNC) is the official data collection of all new cancer cases diagnosed and/or treated in Luxembourg, for both residents and non-residents, in order to have an overview of all cancer patients being cared for in Luxembourg (**Table 1**). The RNC was established to monitor cancer incidence, mortality, and survival trends, to assess the effectiveness of treatments offered to patients, and to evaluate the efficiency of public health prevention efforts, screening programs, and to compare outcomes at international level (16). It also serves as an infrastructure to support epidemiological and clinical cancer research, aligning with national goals to enhance translational and precision cancer research (17). Created in 2013, the RNC is one of Europe’s youngest PBCR. The RNC facilitates national and international comparisons and supports public authorities to plan and tailor health services aligned to the identified needs of the population.

**Table 1:** Example of data items collected by the RNC.

Data type	Examples of data items collected
Record Identification	- Registry Identification Number
Demographic data	- Age at diagnosis - Gender - Country of birth - Last known address
Death data	- Date of death - Cause of death - Autopsy
Tumor data	- Date of incidence - Topography (*ICD-O-3) - Morphology (ICD-O-3) - Basis of diagnosis - Clinical stages - Pathological stages - Metastases at time of diagnosis - Biopsy related data - Cytology related data - Histological prognostic factors - Tissue tumor markers and molecular alterations
Clinical data	- Circumstances of discovery - Comorbidity - Performance score (*ECOG)
Therapeutic management data	- Initial treatment - Surgery - Chemotherapy - Hormone therapy - Radiotherapy - Targeted therapy - Other treatments

*ICD-O-3, International classification of diseases for oncology (ICD-O) – 3rd edition,

*ECOG, Eastern Cooperative Oncology Group.

Significant benefits and wider research scopes are obtained by linking PBCR data with appropriate secondary datasets such as biobanking data, socioeconomic data, or genomic datasets (18). In general, patients’ data routinely collected for a variety of sources other than traditional clinical trials (Real-World Data RWD) can offer a significant potential for cancer research. These sources include: electronic records, administrative claims, disease registries, screening programs, vital statistics, or even wearable digital health tools (19). As RWD can come from different sources and in different formats, it can also facilitate the characterization of health care provision, including health insurances, health providers and patients’ characteristics (20).

These potential linkages enable a broader range of research questions and highly efficient use of PBCR data in cancer control and public health initiatives (21, 22). Some of the limitations of PBCR may include lack of information on longitudinal treatments, drug use, risk factors, comorbidities, quality of care and outcomes other than death. Linking cancer registries with other complementary data sources may provide a more comprehensive picture of disease development and management (23, 24).

The feasibility of data linkage relies on multiple factors including data availability, quality, and completeness of identifying information across data sources (25, 26). Data linkage methods are mainly categorized as deterministic or probabilistic, emphasizing the protection of patient privacy and ensuring compliance with confidentiality regulations (27).

In Luxembourg, potential secondary datasets may come from the Integrated Biobank of Luxembourg (IBBL) which offers biobanking services, including collecting, processing, analyzing, and storing biological samples and associated data (28), as well as the General Inspectorate of Social Security (IGSS), responsible for managing the national health insurance registry (29). Linking data from such sources with RNC may enhance cancer research, particularly in studies focusing on the socioeconomic characteristics of the population.

This study aims to identify opportunities for data linkage between cancer registries and secondary data sources, while outlining the current situation of the RNC. Ultimately, steps for linkages between cancer registries and biobanks and/or sociodemographic data are assessed to enhance cancer research and public health initiatives. To achieve this, a scoping review was conducted to explore published data linkage methodologies, ensuring data confidentiality, and to examine future opportunities for Luxembourg.

In our review, we focus on the questions:

- What are the possibilities for data linkage between cancer registries and other data sources?
- How RNC data linkage with other data sources would contribute to improving cancer research in Luxembourg?
- What methodologies are available for carrying out data linkages ensuring data confidentiality?

## Materials and methods

### A scoping review: examining published methodologies

A scoping review using PubMed and Embase databases was performed utilizing the methodology developed by Arksey and O’Malley, and followed by the backward snowballing search approach in Google Scholar (30, 31). To structure our approach in addressing the research questions, we followed the Preferred Reporting Items for Systematic Reviews and Meta-Analyses extension for Scoping Reviews (PRISMA-ScR) guidelines (32). Selection criteria were developed using the Population, Concept and Context approach as recommended by PRISMA (ScR). The population of this scoping review encompassed all cancer patients from all types of cancer registries (PBCR, hospital based cancer registries, and site specific registries). The main concept was data linkage performed between cancer registries and other data sources such as biobanks, sociodemographic surveys, census data or insurance claims. Our interest was primarily in data linkage methodologies and its feasibility on large scale population based studies. At last, for this review the context was to investigate which kind of data sources would be linkable to Luxembourg's RNC and how new linkages can be used to uncovering new insights for cancer research and enhancing public health interventions.

Therefore, the following algorithm was developed: (“Cancer registry” OR “Population-based cancer registry” OR “Cancer registries” OR “Population-based cancer registries”) AND (Record link* OR Date link* [MeSH]) AND ((Biobanks OR biorepositories) OR (Sociodemographic OR “Census data”)).

The same keywords were used for the backward snowballing approach to create a starter set for the search in Google Scholar. Afterwards, titles of articles from the reference lists were scanned, and pursued to assess the inclusion of relevant articles after full-text reading (30). We conduct a first search in June 2019 and an update in February 2025.

Inclusion criteria were restricted to English-language guidelines, reports, and qualitative and quantitative studies. Articles that did not describe data linkage were excluded. There were no restrictions imposed based on to geolocation and year.

For the selected studies, we extracted the information: authors, year, reference type, country of origin, data source, and title.

## Results

**Figure 1:**
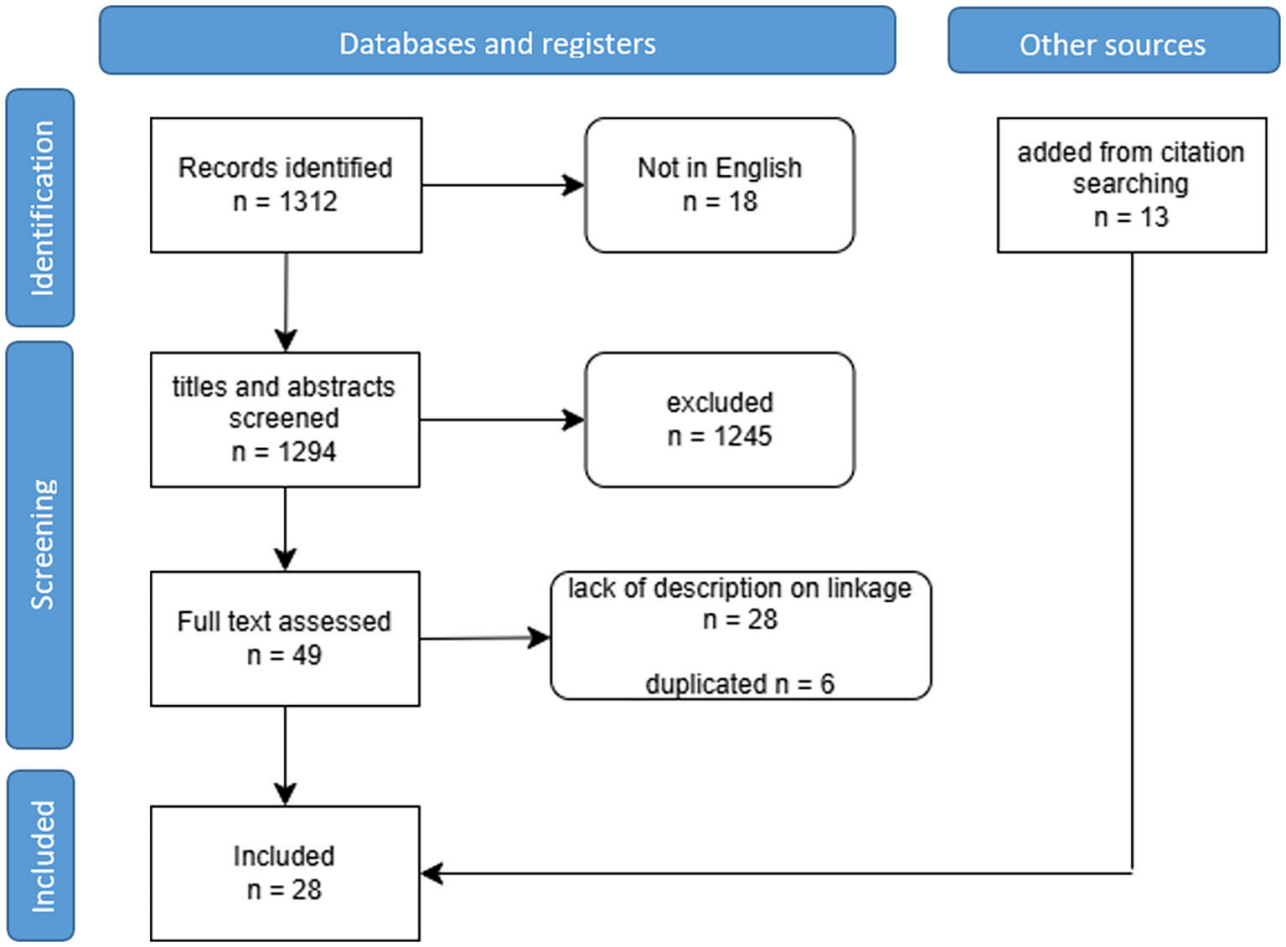
Flowchart of the scoping review process.

In total, 1312 articles were initially identified, including 516 from PubMed and 796 from Embase. After scanning the titles and abstracts, 1245 articles were excluded, leaving 49 for full-text reading. Among these, six duplicate articles and 28 articles lacking data linkage descriptions were further excluded. Remaining fifteen articles (n=15) met the inclusion criteria (**Figure 1**).

**Figure 2:**
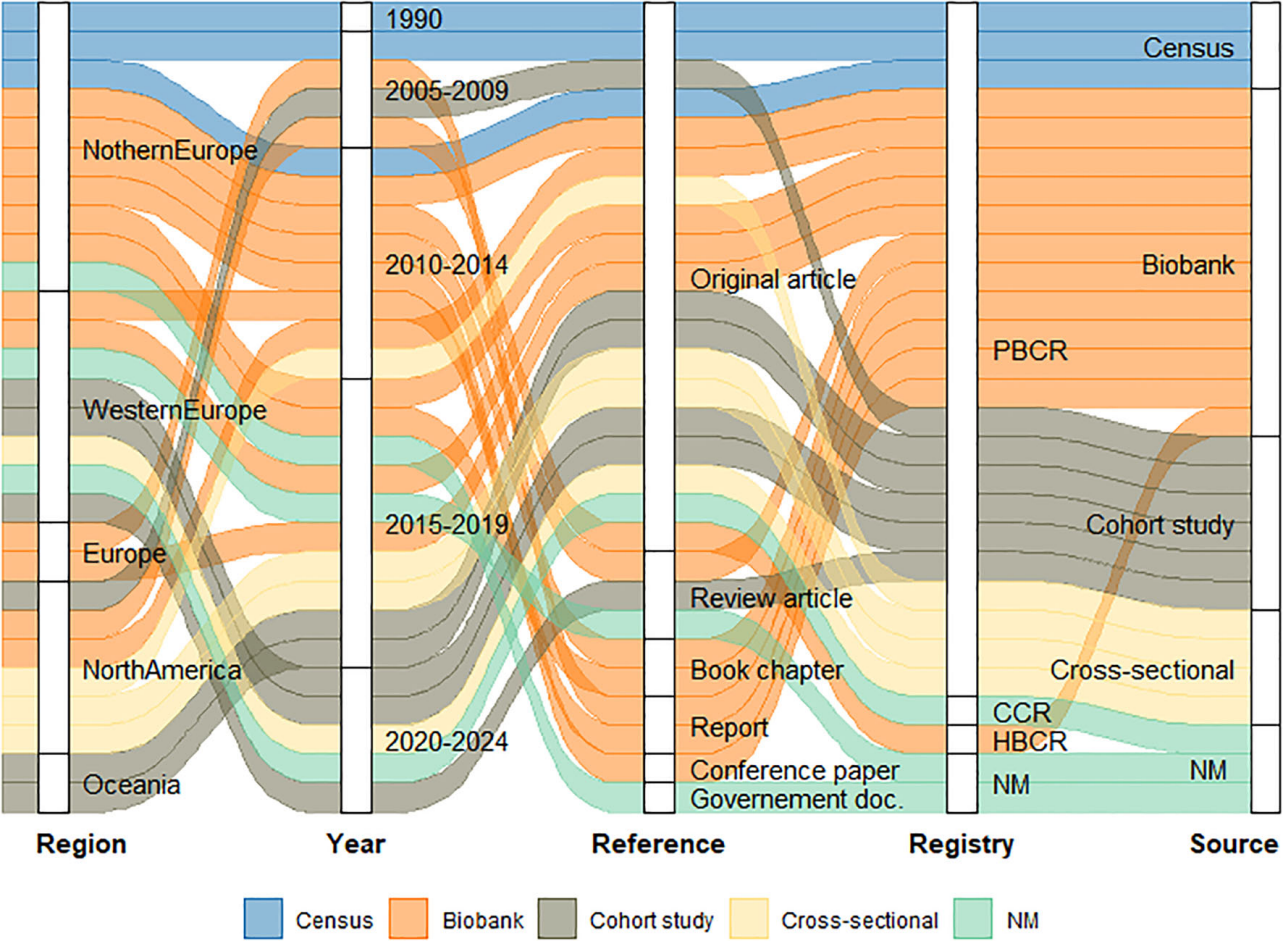
Scoping review results scheme. CCR – Clinical Cancer Registration; HBCR – Hospital Based Cancer Registry; PBCR – Population Based Cancer Registry; NM – Not Mentioned.

Additionally, 13 articles were included by using the snowballing search approach, resulting in 28 articles identified (**Supplementary Table**). All 28 included articles involved cancer registries and examined data linkage at national or regional level. Studies were published between 1990 and 2022, and originated from various countries, including European countries (n=20) (33–47), the USA (n=6) (48–52), and Australia (n=2) (53) (**Figure 2**).

Among the included articles, 12 assessed data linkage between PBCRs and biobanks (33–35, 38, 40, 42–46, 49, 52), and fourteen explored data linkage between PBCRs and administrative, sociodemographic, and other health data (22, 36, 37, 41, 48, 50, 51, 53–59). Two articles described the linkage methodologies used, without specifying the source of the data (39, 47).

Findings were grouped into four themes: data availability and harmonization, addressing heterogeneity and interoperability; data confidentiality, covering legal and ethical requirements; data access and exchange, exploring centralized and federated models; and data linkage methods, outlining deterministic and probabilistic approaches. Within this framework, this scoping review aimed to understand how different studies have approached common obstacles for data linkage.

### Data availability and harmonization

According to a large European study published in 2016, the ability to search for and get access to available data from samples stored at biobanks is essential to combine biomedical and clinical data (43). However, privacy, semantics (vocabularies), and technical heterogeneity challenges the search process for the sample’s information. To formalize and evaluate a methodological framework, the Sample avAILability (SAIL) method for data linkage between the Swedish biobank at Karolinska Institute and the Swedish national prostate cancer registry was used. SAIL operates on availability data (metadata; description of available data), which provides access to summary content at individual records level without disclosing its value, thus temporarily avoiding privacy issues. Retrospective data was harmonized by creating standardized vocabularies, data mapping, and integration. The SAIL web-based system provides an interface for the harmonization and submission of samples or phenotypic data. Furthermore, it is also a search tool to identify suitable cohort data for specific needs (46).

Similar challenges arising from datasets heterogeneity were described in a German study examining an established network between different medical institutions to facilitate translational cancer research architecture (47). To overcome these challenges, the study suggested collaborations among data sources to understand data elements, including vocabulary and semantics, and advocated for the creation of a centralized metadata repository (e.g., a data dictionary) accessible to all partners. This common framework was accompanied by tools supporting remote access and federated analysis, with limited data transfers and strict access control for local partners, promoting trust and data ownership.

### Data confidentiality

In Europe, processing and sharing patient’s data, with the exception of anonymized data, need to comply with national legal requirements and ethical guidelines, such as the European General Data Protection Regulation (GDPR) and country specific national laws (27, 47). In the United States, the privacy rules of the Health and Insurance Portability and Accountability Act (HIPAA) allow access to protected health information without patients permission if the provided information has been previously de-identified to prevent personal identifiers (52).

For biomedical research utilizing data from biobanks, related permissions from national data protection authorities, national or local ethical committees and from the biobank’s organization are needed, often including the requirement of informed consent (35). However, basic consent standards and conditions vary widely depending on respective national laws. Legal requirements for PBCR linkage with sociodemographic data were only briefly discussed in included studies, but clearance by an ethics board was a prerequisite in all cases.

Patients’ consent forms significantly differed between PBCR linkage protocols for biobanks or sociodemographic data.

The extent of patients informed consent could be defined as infinite, broad, or limited (60). Here are examples of consent practices observed in different countries:

1. According to the Belgian biobanks law, all in-patients are informed, thanks to the “hospital welcome brochure” about the potential use of their residual tissue for scientific research. Such a “presumed consent,” turns into informed consent after explicit explanation and document signing (33).
2. According to the Estonian Human Genes Research Act, biobank participants sign a “broad consent,” which permits to use collected samples for research, without previously identifying a specific project, and to retrieve additional participants’ information from other databases (61). The “broad consent” was also been explored in Ireland. Following the recommendations of the Biobanks Irish Trust (BIT), the biobank consent was modified to include provisions for long-term storage and dissemination of both samples and de-identified data (38, 42).
3. According to the Finnish Personal Data Act, collecting and sharing health and social information are allowed only based on a patient’s informed agreement unless the data is collected to be used in statistics, science, or historical research. Moreover, previously collected health data might be used for research without informed consent if the data is extensive or if seeking for informed consent is not possible. However, combining biological samples with registered data requires mandatory ethical board approval (35).
4. In the United States, linking sociodemographic data from the National Center of Health Statistics (NCHS) with cancer registry data requires approval from the NCHS ethics review board and the state’s Department of Health Institutional Review Board (48). Similar requirements apply in Australia, where approval from the Population and Health Services Research Ethics Committee is mandatory to link cancer registry data with population sociodemographic data (53).

### Data access and exchange

Data linkage is challenging in terms of data access and exchange due to confidentiality related restrictions. To facilitate data sharing both horizontal and vertical types of data integration can be employed (62). Horizontal integration includes data from different institutions that cover the same type of data:

- Interconnection between data sources was observed in Belgium for the virtual tumor bank, where all local biobanks were linked to a central database integrated within the Belgian National Cancer Registry. Biobanks export their local data in a standardized template to be uploaded in the central database. Upon request, the linkage with the PBCR is done by the cancer registry staff who has the authority to access that database and search across the biobanks datasets (33).
- In a study from California, PBCR data linkage with biobanks was conducted to establish a virtual biobank (49). Californian biobank data were extracted to Excel files and securely linked with the California Cancer Registry behind the cancer registry’s firewalls.

In contrast, vertical integration combines different types of data:

- A decentralized system or federated system refers to the connection between heterogeneous databases from multiple sources into a unified framework, while the data is kept at its original source under the data owner’s control, with the linkage occurring only when requested. This model was implemented in Estonia, where a national IT architecture was established, supported by a software called “X road”. This open-source ecosystem solution enables unified and secured data exchange between different data services, through which registries and institutions can communicate safely (61). The German Cancer Consortium also explored a similar concept. Here an integration layer, “bridgehead”, is provided for each data source to form a bridge between consortium members, thus creating a harmonized view of all data sources while data owners retain control over data access and are actively involved in all inquiries (47).
- A centralized model, named as data bank or data repository and analyzed in the United Kingdom, stores anonymized data from different sources in a single location. The Secure Anonymized Information Linkage (SAIL) Databank was established to store data from different health and non-health sources, with all data de-identified by a Trusted Third Party (TTP) - in this case, the National Health Service (NHS) (27). A linkage performed by a TTP enables the separation of the linkage and analysis processes, thereby ensuring patient privacy and enhancing data security (22).
- In other scenarios, exists a partnership agreement between cancer registries and biobanks (38, 42). For example, for data linkage between Northern Ireland Cancer Registry and the Northern Ireland Biobank, a full time staff member funded by the biobank is based at the cancer registry to facilitate data linkage procedures.
- For data linkage activities involving national statistics data, a Danish and a Lithuanian study report that the national sociodemographic data was preserved at the statistics governmental department and was not allowed to be exported out of its source (36, 37). The linkage process was mainly performed at the statistics bureau in a secure environment.

A main challenge in designing a data flow for linkage involving personal identifiers is to ensure that end users do not have access to these identifiers and that sensitive information is not transferred to institutions that also possess identifiable data, thereby mitigating the risk of re-identification. However, the quality of the final data linkage remains a critical concern. It is therefore essential to determine the most appropriate format for sharing encrypted identifiers, recognizing that linkage based on non-numeric identifiers carries a higher risk of mismatches (57).

### Data linkage methods

This scoping review identified the following data linkage methodologies, depending on the use of personal identifiable data to assess matching between pairs of records: deterministic record linkage (DRL); probabilistic record linkage (PRL); or a combination of both. PRL involves assigning weight record pairs and assessing the likelihood of these records representing a true match (45). This approach can be implemented sequentially, beginning with the specification of blocking variables, followed by the application of matching variables. Record pairs are retained only if their similarity score surpasses a predefined threshold (55).

The choice of the appropriate linkage method depends on the datasets, available variables and quality regarding accuracy, stability, and completeness (63). DRL uses a linkage key as a single reliable identifier [e.g., a unique personal identifier (34, 36, 41, 44) or Social Security Identification Number (SSIN) (33)] and here all linkage variables used are equally important. In case of absence, a linkage key can be created, by combining linking variables (e.g. first name, last name, date of birth, gender). The linkage key must be produced by an authorized staff from the data source or by a trusted third-party associate (52).

Several studies carried out a two-step data linkage approach combining DRL and PRL. For example, first is checked whether it is possible to make a direct match with an unique identifier (if it exists); otherwise a probabilistic linkage procedure is applied using variables such as name and date of birth (59). Other studies applied only PRL, using various software programs such as Link Plus, developed by the United States Center for Disease Control and Prevention (64), and Choicemaker (65), used by the Centre for Health Record Linkage in Wales, Australia (48, 51, 53, 54).

In Germany, cohort data are typically linked to cancer registry records using a pseudonymisation key based on name, sex, date of birth, and place of residence. A trusted center generates tokens from personal identifiers, which are then matched using probabilistic linkage. Only non-identifiable data are returned to the requesting institution. This probabilistic linkage process is subject to two main types of error: homonym errors, where records from two different individuals are erroneously linked, and synonym errors, where records belonging to the same individual are incorrectly treated as separate entities (56).

Another German study is also worth mentioning. This study employed a DRL using common indirect identifiers between insurance claims and cancer registries. This approach was found to be less expensive and faster than usual PRL. The authors concluded that, although it is possible to use a deterministic linkage with indirect identifiers, the sensitivity of this method is very low and recommended using standard probabilistic methods instead (58).

### Key questions for PBCR data linkage with secondary data sources

**Figure 3:**
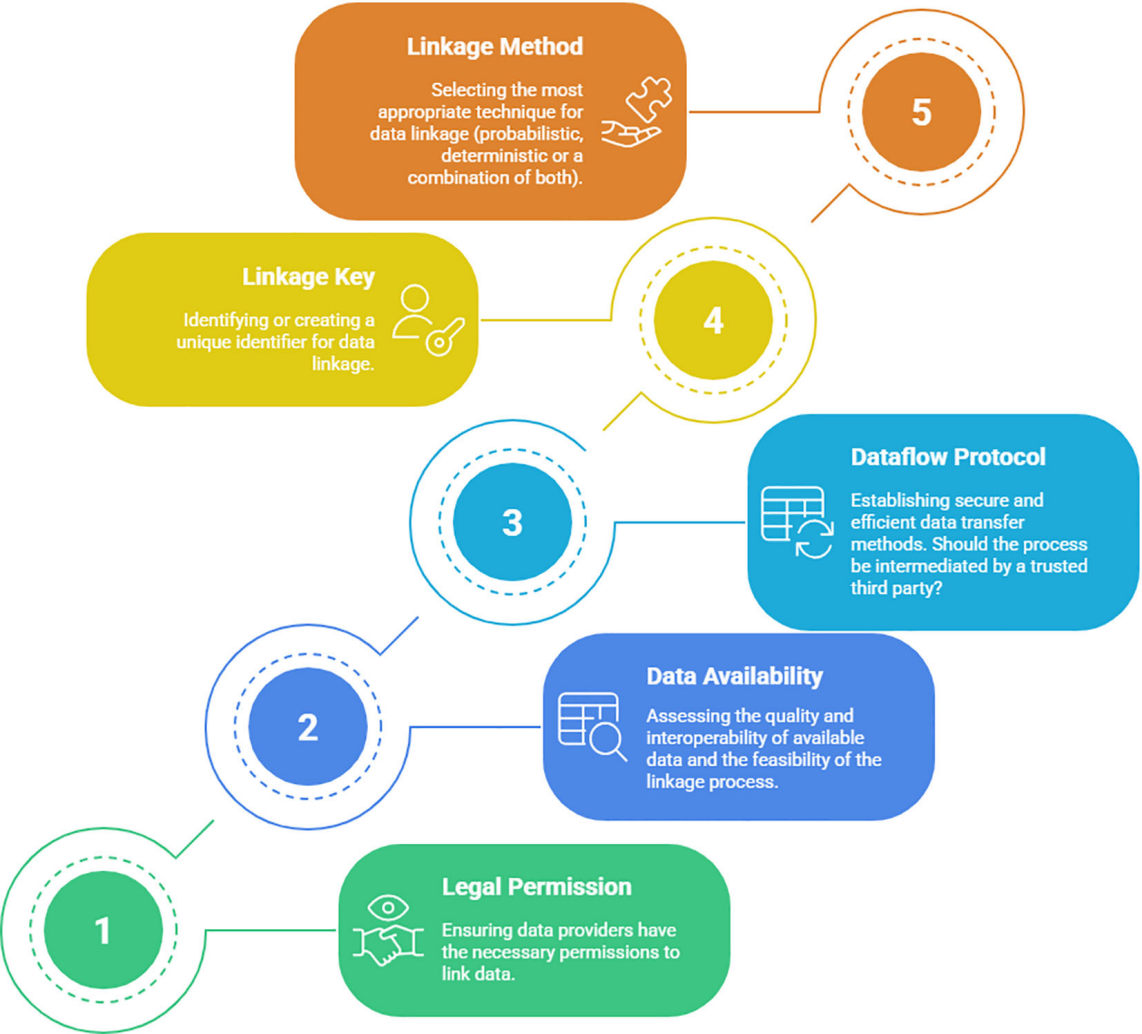
Five key domains and related questions to be considered, step by step (1 to 5), prior to establishing data linkage.

Based on the results of this scoping review, key questions were identified as crucial for establishing data linkage between PBCR and secondary data sources. These questions are categorized into five domains: legal permission, data availability, dataflow protocol, linkage key, and linkage method (**Figure 3**).

Domain 1 - Legal permission: Understanding the legal framework and obtaining the necessary permissions and approvals for data linkage activities are crucial to ensure compliance with regulations and protect individuals’ privacy.

Following questions are formulated:

- Do data providers have the permission to link their data?
- What approval processes are required for data linkage activities?

Domain 2 - Data availability assessment: Assessing the availability and accessibility of data in secondary sources is essential to ascertain the feasibility of data linkage and to identify potential data gaps that may impact the research outcomes.

Following questions are formulated:

- Is a general description of the data available to assess the feasibility of the linkage process? What is the quality of available data? Are datasets interoperable?
- Are data protection, data quality and data use assured and clear for all parties?

Domain 3 - Data flow protocol: Developing a robust data flow protocol is needed to establish efficient and secure processes for data exchange between PBCRs and secondary sources, which will ensure data integrity and confidentiality throughout the linkage process.

Following questions are formulated:

- Is it possible to export and transfer data at an individual level to external data sources? How will the dataflow be performed?
- Is virtual data transfer an option? Does the process need an intermediary acting as a trusted third party?

Domain 4 -Linkage key: Choosing an appropriate linkage key, whether it is a unique identifier or a combination of variables, is critical for accurately matching records between PBCRs and secondary sources, ensuring reliable linkage results.

Following questions are formulated:

- Is there a common unique identifier available for use, or should be created a linkage key?
- Does the process require the involvement of a trusted third party?

Domain 5- Linkage method: Picking the most suitable linkage method, such as DRL, PRL, or a combination of both, depends on the quality and characteristics of the available datasets.

Following questions are formulated:

- Which linking technique (probabilistic, deterministic, or a combination of both) is most suited to apply, given the data quality, researcher’s goals, and available resources?

## Discussion

To the best of our knowledge, this is the first scoping review examining the procedures and methodologies used to link cancer registry data with secondary databases (respective biobank and administrative data sources). Evidence gathered from this scoping review suggests that main challenges in data linkage include high cost processes, privacy issues, dataset heterogeneity, and data quality. Five key domains and their related questions were identified as critical areas requiring clarification before establishing data linkage: legal permission, data availability assessment, dataflow protocol, linkage key, and linkage method.

The earliest study found in the literature review dates back to 1990, indicating that data linkage in the health sector is not a recently discovered activity (37). In this scoping review, we observed that performed linkage steps were rarely described in detail. Studies exploring cancer registry data linkage with biobanks were more extensively analyzed compared to those linking cancer registry data with sociodemographic, administrative, and health data. It is evident from this scoping review that linkage activities and procedures need to be adapted to the context of each country. Methodologies employed differ depending on available resources, the country’s regulations, data quality, and the linkage’s purpose.

According to a Nordic study, the high costs associated with the data linkage process are related to technical setups, data protection procedures (such as consent and ethics committees), or developing key linkage in the lack of a unique national Personal Identity Code (PIC) (60). It was reported that the cost of linking records without PICs can be up to 50 times more expensive than linking records with PICs (35). Data confidentiality should be aligned with local regulations, and participant consent must be obtained when relevant and practical. In addition, a trusted third party can be involved in removing personal identifiers from respective datasets. To overcome dataset heterogeneity, a federated architecture was suggested as a successful approach to facilitate data linkage between heterogeneous data sources in Estonian and German studies (44, 47). However, according to a Swedish study, federated systems proved expensive and frequently impractical due to differences in underlying medical protocols and standard operating procedures (43).

To facilitate data linkage between national and international institutions and between heterogeneous data sources, international efforts and collaborations are essential. PBCRs are multiple source systems that collect all cancer cases in a well-defined geographical area. For decades, they have worked within strong national and international networks, conducting research and using international standards such as the International Classification of Diseases for Oncology (66) and the TNM classification (67), to guarantee quality indicators of completeness, comparability, validity and timeliness (12, 13). However, health data collected from secondary sources may use different standards, definitions, and levels of expertise, posing challenges for its integration. To address this, one of the initiatives to promote large-scale harmonization of health data is being led by the Observational Health Data Sciences and Informatics (OHDSI) consortium (68). Their goal is to facilitate the access to and analysis of health data. For that, they have created the Observational Medical Outcomes Partnership (OMOP) Common Data Model (CDM), a model that aims to standardize the representation of data (format) and its content (terminologies, vocabularies, coding schemes). This model has the capacity to accommodate data from diverse sources such as administrative claims, registries or electronic health records (69). The OHDSI’s OMOP CDM aims to serve as a foundation for federated analytics and to support collaborative research. Supported by the European Health Data & Evidence Network (EHDEN) (70), the RNC and multiple registries and organizations across Europe, as well as others in the United States (US), have explored the OHDSI’s OMOP CDM for a minimal set of variables and assessed its potential to enhance interoperability and support data sharing. Implementing this model may be challenging for some cancer registries with limited IT infrastructure, human resources, or established data-sharing frameworks. Moreover, European and US PBCRs have tested OMOP-CDM for cancer data, reporting improved interoperability but also a loss of data granularity that may limit clinical research. Despite these challenges, the OHDSI’s OMOP CDM ecosystem has proven to be a successful support for cancer research, particularly in large scale collaborative studies (71).

Building on such models can significantly advance precision oncology by enabling the use of high-resolution cancer data to improve diagnosis, treatment, and outcomes. The upcoming European Health Data Space (EHDS) regulation further supports this vision by establishing a unified framework for electronic health data sharing and reuse across the European Union (72), positioning OHDSI’s OMOP CDM initiative as a potential solution for adoption by PBCRs regarding the secondary use of data. However, it may also encourage PBCRs to embrace a different data modelling standard, such as openEHR (73) to support clinical care setting (i.e. primary use of data). Given that the EHDS regulation seeks to enable the reuse of certain data for purposes of public interest and scientific research, while also fostering a dedicated environment for health data within a unified market for digital health products and services, it will promote streamlined and harmonized data linkage within a robust regulatory environment.

Some countries have already introduced legislation to facilitate data linkage by enabling the usage of a PIC. The Nordic Occupational Cancer Study (NOCCA) is a prime example of a high-impact cohort study that collaborates across Nordic countries and links census data with PBCR data, transcending national boundaries to advance larger-scale research. This cohort study provides comprehensive insights into cancer incidence spanning up to 45 years, with a focus on occupational categories within Nordic populations. The study achieved this by linking individual records extracted from census data using the PIC utilized in all Nordic countries (41, 74).

In addition, the EUROCOURSE (Europe against Cancer: Optimization of Use of Registries for Scientific Excellence in Research) project has put forth guidelines, specifically within work package 7, to facilitate the linkage between cancer registries and biobanks. These guidelines are particularly valuable for translational cancer epidemiology and clinical research (75). To enable international operations, the European bio-banking platform (BBMRI) and EUROCOURSE have collaborated in developing a standardized minimum dataset for linking biobanks and cancer registries. This strategy advocates for a cost-effective and relatively uncomplicated approach that can be carried out annually while meeting the scientific expectations of researches (76). This work was pursued by the iPAAC (Innovative Partnership for Action Against Cancer), including in work package 7 the aim to advance PBCRs information to better support evidence-based cancer surveillance and care (77).

In Luxembourg, the second Plan National du Cancer (PNC2) (78) prioritizes, among other objectives, the digitalization and interoperability of health data, the expansion and integration of oncology data systems, the organization of oncology services into specialized competence networks, and the advancement of translational cancer research. In alignment with these objectives, and supported by the PNC2, the RELIANCE study, or “REaL-life cANCEr epidemiology to identify risk factors for cancer with a particular focus on prevention and care” (79), aims to evaluate cancer epidemiology for the first time in Luxembourg using longitudinal population data from the RNC. In addition, the RELIANCE study will investigate a range of research questions using RNC data, as well as exploring potential secondary data linkages with the RNC data. While the study is likely to expand to include different cancer sites in future, the first pilot study (RELIANCE – Breast Cancer) aims to evaluate breast cancer epidemiology in the Grand Duchy of Luxembourg.

Evidence selection bias may have been introduced because of the decision to restrict the study’s search strategy to the PubMed and Embase databases and use a backward snowballing approach on Google Scholar (80). However, the scoping review only examined the linkage between PBCRs and biobanks and/or administrative or sociodemographic databases. This provides valuable insights into the linkage activities with respective sources. Although, a wider systematic review is required, including other potential secondary data sources (e.g., electronic health records, institutional or organizational databases, and several others).

Nevertheless, this study has laid the foundation for formulating a more targeted research question that emphasizes the linkage between PBCRs and prospective secondary data sources. This focus is particularly valuable for population-based studies, as it enables researchers to prioritize and explore the linkage activities and potential benefits associated with combining PBCR data with other datasets.

## Perspectives and conclusions

The current state of cancer prevention, treatment, and care calls for a renewed commitment and adaptation to the rapid progress in oncology (51). The European Action Plan Against Cancer emphasizes the importance of addressing the entire cancer pathway. In this context, the RNC explores and evaluate existing secondary data sources and provides innovative solutions that meet the current cancer-related needs of the population. Moreover, the RNC actively support novel cancer research through digitalization, innovation, and interprofessional collaboration, to contribute to the development and implementation of comprehensive cancer control initiatives (81).

To enhance cancer research and innovation, RNC explores data linkage with suitable secondary data sources. The reviewed literature demonstrated the viability of achieving such a linkage when key questions are solved. These linkage efforts enhance the potential of cancer registries and biobank or socioeconomic databases by widening their research opportunities. Furthermore, to overcome the heterogeneity in datasets, RNC has applied OHDSI’s OMOP CDM and successfully started the RELIANCE study. While data linkage can be performed independently of any data model, CDM improves data interoperability and facilitates the linkage process with other data sources in the same format. Potentially, this may lead to the use of clinical and epidemiological cancer data to improve patient outcomes both nationally and internationally.

PBCRs often face constraints in the range of data items collected for hypothesis-driven research, and collecting new variables can be time-consuming, costly, and susceptible to bias (82). Therefore, a promising avenue for future research involves linking PBCR data with population-based health surveys such as EHES-LUX, ORISCAV-LUX (waves 1 and 2) or similar surveys (51, 83, 84). Another illustrative example come from a Norwegian study that successfully demonstrated the linkage of a PBCR with a health interview survey to investigate cardiovascular risk factors in different cancer types (85). Linking cancer registry data with health interview surveys enables clinical cancer registry variables to be evaluated alongside individual risk factors, socioeconomic position, screening behaviors, and healthcare utilization variables, which are typically unavailable in cancer registries. Further research is needed to assess the feasibility and true potential of such linkages, but the prospects are promising, providing ample scope for future investigations. To move beyond *ad hoc* and project based initiatives, RNC data linkages should be formalized as part of a long-term national strategy. However, achieving this requires dedicated funding, clear governance structures and standardized procedures to ensure continuity, interoperability, and scalability over time.

The quality of PBCRs’ data is generally assessed across four dimensions (12, 13). Comparability refers to the extent to which coding, classification, data recording and reporting definitions comply with international guidelines. Validity is defined as the proportion of cases in the registry that actually have a given characteristic. Timeliness measures how long it takes for registry information to be made available to professionals and researchers. Completeness is defined as the extent to which all incident cancers occurring in the population are included in the registry database. Linked datasets must also be evaluated according to these criteria to ensure that linkage itself does not compromise data integrity neither the quality of the PBCR data in any of its dimensions.

Strengthening linkage activities of PBCRs with secondary sources offers substantial benefits, enabling a more comprehensive exploration of cancer prevention, diagnosis, treatment, and prognostic factors. The findings of this study offer valuable insights into key questions that need to be addressed before establishing data linkage between PBCRs and secondary sources.

By addressing the key questions identified herein and considering the five aforementioned domains, researchers and policymakers can establish robust and effective data linkage strategies, thereby unlocking the potential of PBCRs and facilitating valuable research on cancer-related topics.
