# Supplementary material for: Optimizing data linkage for maximizing the potential of Luxembourg’s national cancer registry: a comprehensive scoping review
Source: Front Oncol. 2025 Nov 18;15:1679408. doi: 10.3389/fonc.2025.1679408 (PMC12668946; doi:10.3389/fonc.2025.1679408)
Supplement: Supplementary file 1 [file Table1.docx]

Supplementary Material

# Supplementary Table: Summary of included articles.

| Author | Reference Type | Study origin | Data source | Title |
| --- | --- | --- | --- | --- |
| Kim Vande Loock et al., 2019 | Journal; Frontiers in Medicine | Belgium | Biobank | The Belgian Virtual Tumor bank: A Tool for Translational Cancer Research |
| Hilde Langseth et al., 2010 | Journal; Acta Oncologica | Nordic countries | Biobank | Ensuring quality in studies linking cancer registries and biobanks |
| Eero Pukkala, 2011 | Methods in molecular biology (Clifton, N.J.) | Nordic countries | Biobank | Biobanks and Registers in Epidemiologic Research  on Cancer |
| Miller EA et al., 2014 | Journal; Vital and health statistics. Series 2, Data evaluation and methods research | United States of America | Cross-sectional household survey | Linkage of 1986–2009  National Health Interview  Survey With 1981–2010  Florida Cancer Data System |
| Nicola Creighton et al., 2018 | Journal; BMC Medical Research Methodology | Australia | Cohort study | Self-selection in a population-based cohort  study: impact on health service use and survival for bowel and lung cancer  assessed using data linkage |
| Margaret E. McCusker et al., 2012 | Journal; BIOPRESERVATION AND BIOBANKING | United States of America | Biobank | Feasibility of Linking Population-Based Cancer  Registries and Cancer Center Biorepositories |
| Giedre Smailyte et al., 2013 | Journal; Cancer Epidemiology | Lithuania | Census records | Suicides among cancer patients in Lithuania: A population-based census-linked study |
| Pascal Guenel et al., 1990 | Journal; British Journal of Industrial Medicine | Denmark | Census records | Laryngeal cancer in Denmark: a nationwide  longitudinal study based on register linkage data |
| Claire Lewis et al., 2018 | Journal; Open Journal of Bioresources | Ireland | Biobanks | The Northern Ireland Biobank: A Cancer Focused Repository of Science |
| Blanaid Mee et al., 2013 | Journal; BIOPRESERVATION AND BIOBANKING | Ireland | Biobank | Development and Progress of Ireland’s Biobank Network: Ethical, Legal, and Social Implications (ELSI), Standardized Documentation, Sample  and Data Release, and International Perspective |
| Ola Spjuth et al., 2016 | Journal; European Journal of Human Genetics | Europe | Biobank | Harmonizing and linking biomedical and clinical data across disparate data archives to enable integrative cross-biobank research |
| Martin Lablans et al., 2018 | Journal; American Society of Clinical Oncology | Germany | NA | An Architecture for Translational Cancer  Research As Exemplified by German  Cancer Consortium |
| Ola Spjuth et al., 2014 | Conference proceedings | Sweden | Biobank | Data Integration between Swedish National Clinical Health Registries and Biobanks Using an Availability System |
| Adelaide Ariel et al., 2014 | Report | Netherlands | Biobank | Record Linkage in Health  Data: a simulation study |
| Liis Leitsalu and Andres Metspalu, 2017 | Book chapter | Estonia | Biobank | From Biobanking to Precision Medicine: Estonian Experience |
| Limin X. Clegg et al., 2009 | Journal; Cancer Causes Control | United States of America | Cohort study | Impact of socioeconomic status on cancer incidence and stage  at diagnosis: selected findings from surveillance, epidemiology,  and end results: National Longitudinal Mortality Study |
| Laura A. McClure et al., 2016 | Journal; Journal of registry management | United States of America | Cross-sectional household survey | Linking National Health Interview Survey with Florida  Cancer Data System: A Pilot Study |
| Eero Pukkala et al., 2009 | Journal; Acta Oncologica | Nordic countries | Census records | Occupation and cancer – follow-up of 15 million  people in five Nordic countries |
| Rajiv Dhir et al., 2008 | Journal; Cancer | United States of America | Biobank | A multi-disciplinary approach to honest broker services for  tissue banks and clinical data: a pragmatic and practical model |
| Kerina Jones and David Ford, 2018 | Government document | United Kingdom | NA | Privacy, confidentiality and practicalities in data linkage |
| Eleni Zika et al., 2008 | Journal; pharmacogenomics | Europe | Biobanks | Sample, data use and protection in biobanking in Europe: legal issues |
|  |  |  |  |  |
| Arndt et al., 2020 | Gesundheitswesen | Europe | Cohort study | Data from Population-based Cancer Registration for Secondary Data Analysis: Methodological Challenges and Perspectives. |
| Heins et al., 2022 | BMC Medical Research Methodology | Europe | Cohort study | Opportunities and obstacles in linking large health care registries: the primary secondary cancer care registry ‑ breast cancer |
| Gallaway et al., 2019 | JCO clinical cancer informatics | United States of America | Cross-sectional | Identifying Smoking Status and Smoking Cessation Using a Data Linkage Between the Kentucky Cancer Registry and Health Claims Data |
| Subramaniam et al., 2019 | BMJ open | Australia | Cohort study | Post-colonoscopy colorectal cancers identified by probabilistic and deterministic linkage: results in an Australian prospective cohort |
| Kollhorst et al., 2021 | Pharmacoepidemiology and Drug Safety | Europe | Cohort study | Record linkage of claims and cancer registries data—Evaluation of a deterministic linkage approach based on indirect personal identifiers |
| Langner et al., 2020 | Gesundheitswesen | Europe | Cross-sectional | Linkage of Routine Data to Other Data Sources in Germany: A Practical Example Illustrating Challenges and Solutions |
| Scheel et al., 2021 | Studies in health technology and informatics | Europe | NA | Record Linkage in Clinical Cancer Registration: Experiences and Findings from Lower Saxony |
